# Evolution of Antibiotic Synthesis Gene Clusters in the *Streptomyces globisporus* TFH56, Isolated from Tomato Flower

**DOI:** 10.1534/g3.119.400037

**Published:** 2019-04-24

**Authors:** Gyeongjun Cho, Youn-Sig Kwak

**Affiliations:** * Division of Applied Life Science (BK21Plus), Gyeongsang National University, Jinju 52828, Republic of Korea; † Department of Plant Medicine, Institute of Agriculture & Life Science, Gyeongsang National University, Jinju 52828, Republic of Korea

**Keywords:** *Streptomyces* complete genome, linear chromosome, bioactive compound

## Abstract

*Streptomyces* species are known to produce various bioactive metabolites that can prevent plant diseases. Previously, the *Streptomyces* strain TFH56 was found to inhibit the gray mold pathogen, *Botrytis cinerea*, in tomato flower. In this study, the genome sequence of strain TFH56 was acquired using the Pacific Biosciences RS II platform. Three linear sequences (7.67 Mbp in total) were obtained. Based on average nucleotide identity, strain TFH56 was classified as *Streptomyces globisporus*, which is consistent with the presence of a linear chromosome and linear plasmids. Moreover, as with other examples of *S. globisporus*, the genome of strain TFH56 included a caryolan-1-ol synthase gene, a conprimycin synthetic gene cluster, and a lidamycin synthetic gene cluster

Members of the genus *Streptomyces* are Gram-positive bacteria that resemble filamentous fungi. *Streptomyces* spp. typically inhabit soil, but are also known to interact with plants and animals. The complex life cycle of *Streptomyces* includes spore germination, vegetative hyphal growth, and programmed cell death in vegetative hyphae accompanying the growth of aerial spore-bearing hyphae (Yagüe *et al.* 2013). The genomes of *Streptomyces* spp. have large amounts of G + C content, and some species contain linear chromosomes and plasmids (Bentley *et al.* 2002; Ikeda *et al.* 2003; Li *et al.* 2016). *Streptomyces* spp. are well known as producers of numerous bioactive compounds. According to Bérdy (2012), in 2010 roughly 33,500 bioactive compounds had been isolated from organisms, and 10,400 of these were reported in *Streptomyces* spp. This finding suggests that interactions between *Streptomyces* spp. and other organisms have affected the expression of biosynthetic gene clusters and the evolution and diversity of secondary metabolites. *Streptomyces* spp. have been recognized to interact with >500 other species (Euzeby 2008), and, because of its diversity, the genus *Streptomyces* has been considered as a great potential source of new bioactive compounds. Some *Streptomyces* strains have been identified as antagonists of plant pathogens (Taechowisan *et al.* 2005; Cha *et al.* 2016; Kwon *et al.* 2018). In one study, *Streptomyces* strain TFH56 was isolated from tomato flowers in Jinju, Republic of Korea. This strain was found to be symbiotic with its host in a metagenome study, and showed outstanding antagonism against *Botrytis cinerea*—a causal pathogen of gray mold in tomato (Kwon *et al.* 2018).

The aims of the present study were to identify the known *Streptomyces* species that is related most closely to strain TFH56, to identify genes associated with the biosynthesis of bioactive compounds in this strain, and to clarify the taxonomy and diversity of these genes. These aims were accomplished through complete genome sequencing, followed by the assessment of average nucleotide identity (ANI) with whole genomes of other *Streptomyces* spp., and comparison of the biosynthetic genes in strain TFH56 with their homologs in other *Streptomyces* spp.

## Materials and Methods

### Genome sequencing and identification

Genome sequencing for strain TFH56 was conducted with the Pacific Biosciences RS II platform of Macrogen Inc. (Daejeon, Republic of Korea). The same platform was used to acquire the complete genome of *Streptomyces globisporus* strain S4-7 (Kim *et al.* 2019). Sequence reads were assembled with Canu (version 1.7), and the assembled sequences were polished with the Arrow algorithm in SMRT link (version 5.1.0). The Arrow polish was based on aligned subread BAM files derived from converted bax.h5 files; these served as the raw data files provided by the platform. The genome sequence was annotated via the RASTtk pipeline (Brettin *et al.* 2015) on the RAST web server (version 2.0; http://rast.nmpdr.org/). The RASTtk pipeline was set as the default, and it functioned by turning on domain bacteria and automatically fixed error options. To identify the species of strain TFH56, 16S rRNA sequences of Actinobacteria were analyzed for maximum likelihood after MUSCLE alignment in MEGA X (version 10.0.5). Furthermore, the genome sequence was compared with previously reported genomes from other *Streptomyces* spp. with Pyani (version 0.2.8)—a Python 3 module that can calculate ANI with MUMmer (version 3.23). In this study, 24 complete genome-sequenced strains of Actinobacteria were analyzed. The Ubuntu 18.04 shell commands of Pyani with MUMmer were noted in the Pyani heatmap described in the Supplemental Data. To confirm the ANI results, genome sequences were aligned and visualized with dot plots using DECIPHER (version 2.10.0).

### Comparison of bioactive compound biosynthetic gene clusters

Secondary metabolite biosynthetic gene clusters in strain TFH56 were identified with the bacterial version of antiSMASH 4.1.0 (https://antismash.secondarymetabolites.org/). Homologous regions on each genome were identified using NCBI Blastn (https://blast.ncbi.nlm.nih.gov/). These gene clusters were compared with their homologs in *S. globisporus* via sequence alignment with DECIPHER. The homologous regions on each genome were identified using NCBI Blastn (https://blast.ncbi.nlm.nih.gov/).

### Visualization

Almost all figures were created with ggplot2 (version 3.1.0), gplots (version 3.0.1.1), and DECIPHER in R. The R code is described in detail in Supplemental Material, File S1. The completed figures from R were edited with respect to font size and type, and layout.

### Data availability

Sequence data are available at GenBank; the accession numbers are listed in Table 1. Strains are available upon request. Supplemental material available at FigShare: https://doi.org/10.25387/g3.8030795.

**Table 1:** *Streptomyces globisporus* TFH56 genome assembly and annotation data

Attribute	Chromosome	pTFSG1	pTFSG2	Total
Number of bases	7,488,586	127,820	50,115	7,666,521
Number of assembled reads	16,130	411	54	16,595
Circular	No	No	No	
G + C content	71.6%	69.5%	67.9%	71.5%
CDS	6991	120	61	7172
tRNA	65	1	0	66
rRNA	18	0	0	18
Accession number	CP029361	CP029362	CP029363	

## Results and Discussion

**Figure 1:**
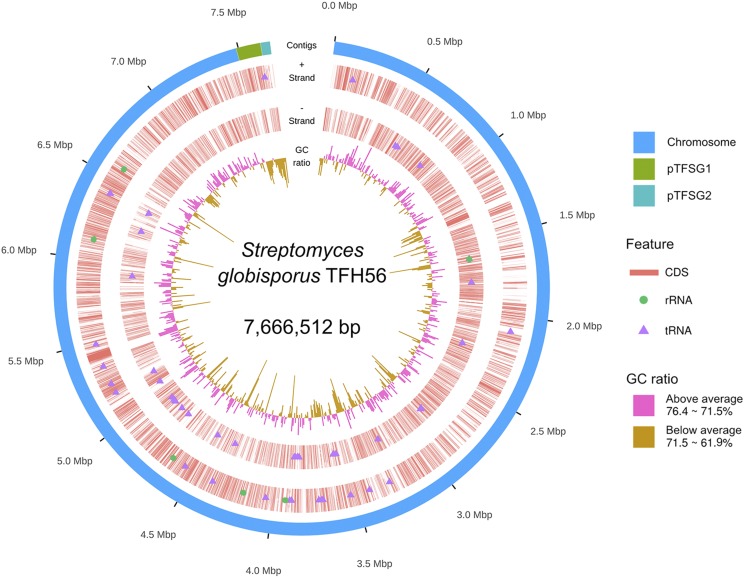
Genome map of *Streptomyces globisporus* TFH56. The outermost open ring shows the linear chromosome and linear plasmids. The innermost open ring shows the GC ratio, calculated from 10,000 bp. The remaining two open rings indicate CDS, rRNA, and tRNA on each strand.

**Figure 2:**
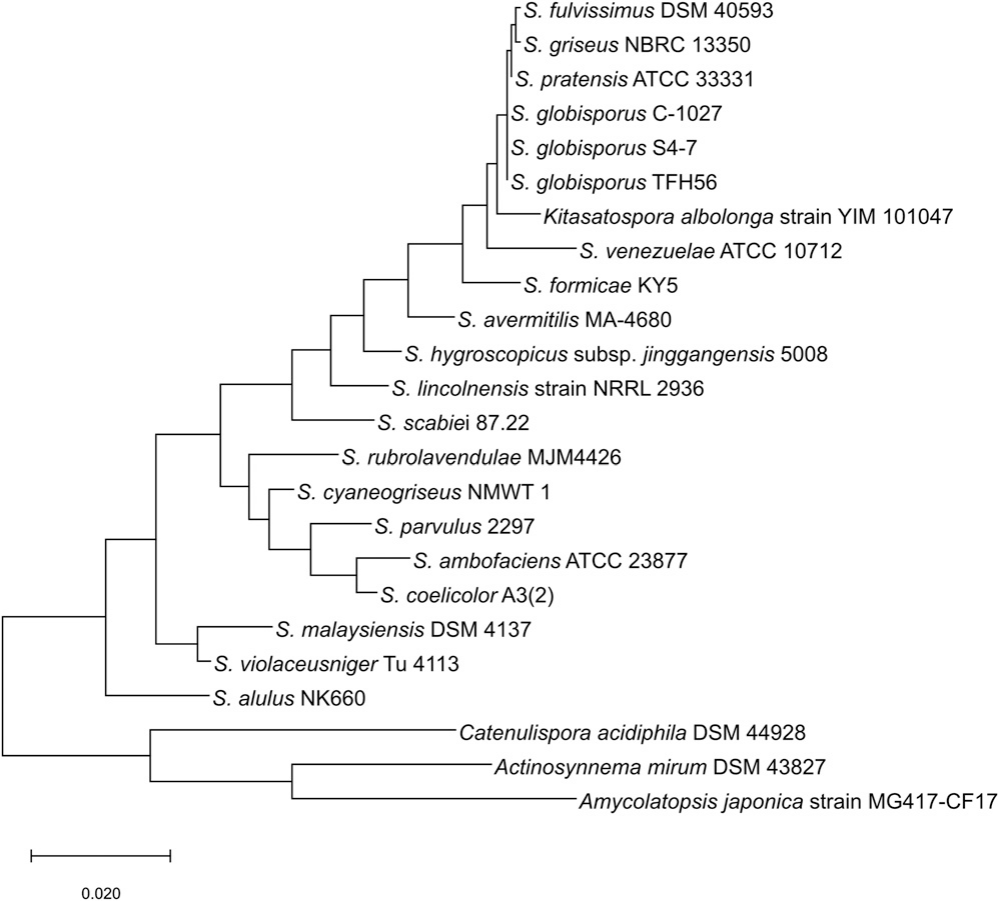
Evolutionary history tree for the 16S rRNA sequence, inferred using the Tamura-Nei mode and maximum likelihood method. The tree involved 24 strains of Actinobacteria, and its sequences are ∼1500 bp. Taxonomically, TFH56 is related most closely to *Streptomyces globisporus*.

**Figure 3:**
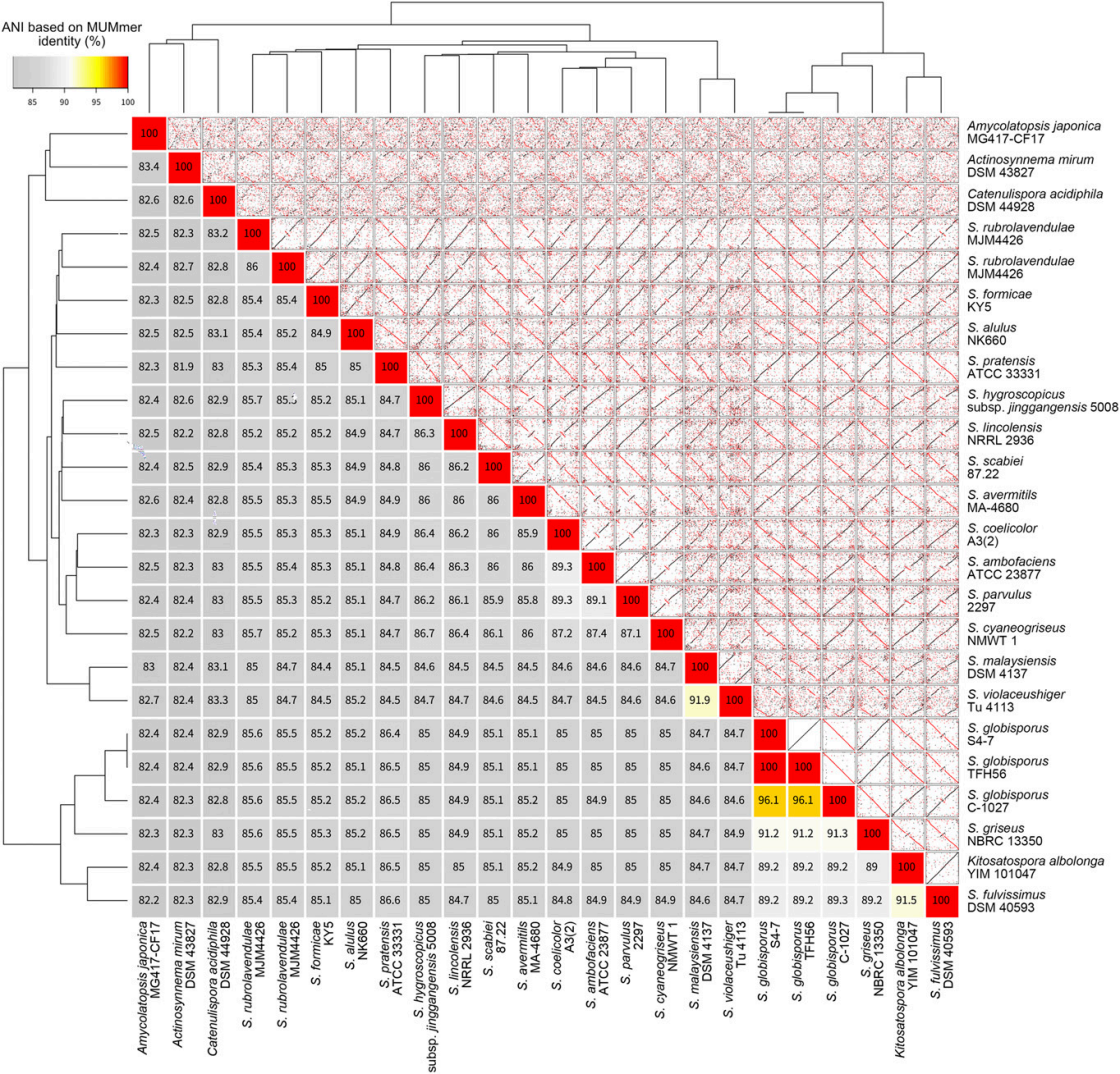
Average nucleotide identity based on MUMmer (ANIm) identification and alignment of 21 *Streptomyces* strains and three Actinobacteria members. The lower left heatmap shows ANIm identity. The trees show similarity according to ANIm identity scores. The upper right subplots are dot plots of whole genome alignments. Black dots represent same-direction alignments, and red dots represent reverse-direction alignments.

This study was designed to acquire a whole genome sequence for strain TFH56, which was isolated from tomato flower, to classify this strain within the genus *Streptomyces*, and to identify genes in strain TFH56 that are homologous to sequences associated with the biosynthesis of bioactive compounds in other Actinobacteria genomes. The acquired genome sequence was assembled in three contigs totaling 7.67 Mbp with 71.5% G + C content, 7172 CDS, 66 tRNA, and 18 rRNA (Figure 1 and Table 1). In evolutionary terms, TFH56 was closest to *S. globisporus* in terms of 16S rRNA (Figure 2). ANI calculations performed with MUMmer indicated that the genome of strain TFH56 had 96.11% sequence similarity with that of *S. globisporus* C-1027, and 99.99% similarity with that of *S. globisporus* S4-7 (Cha *et al.* 2016; Kim *et al.* 2019), which was isolated from strawberry rhizosphere (Figure 3). Unexpectedly, *Kitasatospora* was closely related to *Streptomyces* in the analysis of ANI and 16S rRNA, both of which were associated with the former classification of *Kitasatospora albolonga* as *Streptomyces albolongus* (Labeda *et al.* 2017). Moreover, consistent with the linear chromosome and two linear plasmids (pTFSG1 and pTFSG2) identified in this study, linear chromosomes and plasmids have been reported for *S. globisporus* (Li *et al.* 2016). In strain TFH56, the plasmids had less G + C content than did the chromosome (Figure 1 and Table 1).

antiSMASH predicted 27 secondary metabolite gene clusters in the genome of strain TFH56 (Table 2). Caryolan-1-ol synthase was identified in cluster 9, and a thiopeptide sequence associated with conprimycin biosynthesis in *S. globisporus* S4-7 (Cha *et al.* 2016) was identified in cluster 15. Lidamycin biosynthesis genes were also detected in cluster 27. *S. globisporus* S4-7 is known to produce the antifungal compounds conprimycin (Cha *et al.* 2016) and caryolan-1-ol (Cho *et al.* 2017), and *S. globisporus* C-1027 is known to produce the antitumor agent lidamycin (originally called C-1027) (Hu *et al.* 1988; Liu *et al.* 2002; Li *et al.* 2016). Lidamycin biosynthesis gene sequences could not be found in other *Streptomyces* spp., and sequences associated with caryolan-1-ol and conprimycin biosynthesis could be found only among close relatives of *S. globisporus* based on the ANI phylogeny.

**Table 2:** Secondary metabolite gene clusters in THF56

Cluster	Type	From (bp)	To (bp)	Most similar known cluster
The following clusters are from record chromosome				
Cluster 1	T1pks-Nrps	66,088	117,817	Daptomycin biosynthetic gene cluster (7% of genes show similarity)
Cluster 2	T3pks	208,321	249,373	Tetronasin biosynthetic gene cluster (11% of genes show similarity)
Cluster 3	Melanin	287,733	298,206	Istamycin biosynthetic gene cluster (4% of genes show similarity)
Cluster 4	Bacteriocin-T1pks-Nrps	495,294	568,024	SGR PTMs biosynthetic gene cluster (100% of genes show similarity)
Cluster 5	Terpene	622,086	648,659	Hopene biosynthetic gene cluster (69% of genes show similarity)
Cluster 6	Bacteriocin	1,229,256	1,240,782	—
Cluster 7	T1pks-Nrps	1,269,703	1,347,182	Phosphinothricin biosynthetic gene cluster (6% of genes show similarity)
Cluster 8	Siderophore	1,544,907	1,559,623	—
Cluster 9	Terpene	1,952,004	1,973,011	—
Cluster 10	Ectoine	2,096,983	2,107,357	Pristinamycin biosynthetic gene cluster (23% of genes show similarity)
Cluster 11	Lantipeptide	2,332,022	2,354,832	AmfS biosynthetic gene cluster (100% of genes show similarity)
Cluster 12	T1pks-Nrps	2,427,242	2,481,916	Enduracidin biosynthetic gene cluster (8% of genes show similarity)
Cluster 13	Lantipeptide-Butyrolactone	3,047,239	3,105,744	Labyrinthopeptin A1, A3/labyrinthopeptin A2 biosynthetic gene (40% of genes show similarity)
Cluster 14	Lassopeptide	3,205,276	3,227,973	SRO15-2005 biosynthetic gene cluster (100% of genes show similarity)
Cluster 15	Thiopeptide	4,506,592	4,539,162	Siomycin biosynthetic gene cluster (7% of genes show similarity)
Cluster 16	Nrps	4,781,622	4,826,601	Bottromycin A2 biosynthetic gene cluster (27% of genes show similarity)
Cluster 17	Siderophore	4,937,822	4,949,600	Desferrioxamine B biosynthetic gene cluster (100% of genes show similarity)
Cluster 18	Lantipeptide	5,001,775	5,024,819	—
Cluster 19	Ectoine	6,040,034	6,050,432	Ectoine biosynthetic gene cluster (100% of genes show similarity)
Cluster 20	Terpene	6,475,918	6,497,021	Steffimycin biosynthetic gene cluster (19% of genes show similarity)
Cluster 21	Terpene	6,782,807	6,808,026	Isorenieratene biosynthetic gene cluster (85% of genes show similarity)
Cluster 22	T3pks	7,102,494	7,143,612	Herboxidiene biosynthetic gene cluster (6% of genes show similarity)
Cluster 23	Nrps	7,185,040	7,270,761	Griseobactin biosynthetic gene cluster (100% of genes show similarity)
Cluster 24	Terpene-Nrps	7,290,141	7,356,887	—
Cluster 25	Butyrolactone	7,380,909	7,391,850	Coelimycin biosynthetic gene cluster (16% of genes show similarity)
Cluster 26	Terpene	7,431,928	7,457,471	Isorenieratene biosynthetic gene cluster (100% of genes show similarity)
The following clusters are from pTFSG1				
Cluster 27	T1pks-Nrps	16,475	112,894	Lidamycin biosynthetic gene cluster (93% of genes show similarity)

**Figure 4:**
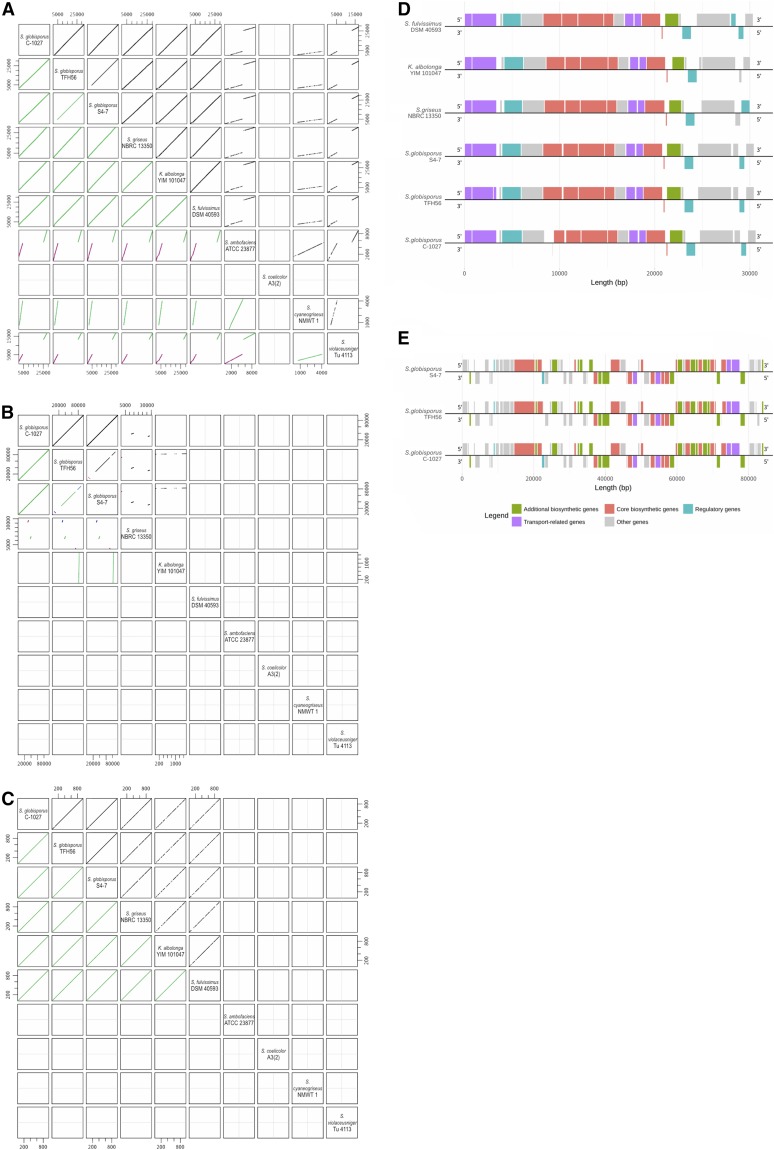
Dot plots of aligned secondary metabolite related sequences and comparisons of their CDS, performed with antiSMASH. (A and D) C-1027 lidamycin synthetic gene cluster and homologs. (B and E) S4-7 conprimycin synthetic gene cluster and homologs. (C) Caryolan-1-ol synthase. All axes at dot plots are lengths of DNA sequences. Black and red diagonals in the upper right block correspond to forward and reverse strands, respectively. In the lower left block, the gradient from green to blue to magenta indicates alignment from the highest to the lowest score. Strain or species axis order depends on the ANI results. In (D and E), each filled block indicates a CDS.

The sequences of these biosynthetic genes were compared among 10 strains in ANI species-similarity order (Figure 4). The caryolan-1-ol synthase sequence in strain TFH56 comprised 978 bp. This sequence was identical to that in strain S4-7, but differed by 24 bp from the sequence in strain C-1027. *Streptomyces griseus* NBRC 13350, *K. albolonga* YIM 101047, and *Streptomyces fulvissimus* DSM 40593 all had similar caryolan-1-ol synthase sequences (Figure 4C). Based on the antiSMASH analyses of CDS structure (Figure 4, D and E), which produced results consistent with the ANI results, the conprimycin biosynthesis sequence in strain TFH56 was 99–100% similar to the sequence in strain S4-7 (Table S1), and was also similar to the homologous sequences in strain C-1027, *S. griseus*, *K. albolonga*, and *S. fulvissimus* (Figure 4, A and D). The homolog of the *S. globisporus* lidamycin biosynthetic cluster in strain TFH56 was located in pTFSG1. This finding corresponded to a previous report that this cluster is located in the linear plasmid SGLP1 in strain C-1027 (Li *et al.* 2016). The lidamycin cluster in strain TFH56 differed from that in strain S4-7 in the 0–25 and >65 kbp regions (Figure 4, B and E). However, the lidamycin CDS structure in strain TFH56 was similar to that in strain C-1027, with the exception of the regulator gene, which differed by roughly 22 kbp in the most similar sequence (Figure 4E and Table S2). These results indicate that genes involved in the biosynthesis of caryolan-1-ol, conprimycin, and lidamycin are unique to *S. globisporus* and its closest relatives among sequenced *Streptomyces* strains (Figure 4 and Figure S1). This finding could improve our understanding of the diversity and evolution of biosynthetic gene clusters among plant probiotic *Streptomyces* spp. strains.
